# Hedonic and Utilitarian Performances as Determinants of Mental Health and Pro-Social Behaviors among Volunteer Tourists

**DOI:** 10.3390/ijerph17186594

**Published:** 2020-09-10

**Authors:** Heesup Han, Bo Meng, Bee-Lia Chua, Hyungseo Bobby Ryu

**Affiliations:** 1 College of Hospitality and Tourism Management, Sejong University, 98 Gunja-Dong, Gwanjin-Gu, Seoul 143-747, Korea; heesup@sejong.ac.kr; 2 Department of Tourism Management, Shanxi University, No. 92 Wucheng Road, Taiyuan 030006, China; bo.meng@sxu.edu.cn; 3 Department of Food Service and Management, Faculty of Food Science and Technology, Universiti Putra Malaysia, Serdang 43400, Malaysia; chuabeelia@upm.edu.my; 4 College of Health Sciences, Food Franchise Department, Kyungnam University, 7 Kyungnamdaehak-Ro, Masanhappo-Gu, Changwon 51767, Korea

**Keywords:** pro-social intention for volunteer tourism, hedonic performance, utilitarian performance, mental health, engagement, problem awareness, ascribed responsibility

## Abstract

International volunteer tourism is an emerging and sustainable trend of the global tourism industry. In this study, we attempted to provide a clear comprehension of volunteer tourists’ mental health increase and pro-social intention formation. A survey method and quantitative approach were used. Our result from the structural analysis showed that hedonic and utilitarian performances, mental health, and volunteer tourism engagement had significant associations and that these relationships contributed to improving pro-social intention. In addition, results from the metric invariance assessment revealed that the volunteer tourism engagement and pro-social intention relation was under the significant influence of problem awareness and ascribed responsibility. Mental health and engagement acted as significant mediators. The comparative importance of volunteer tourism engagement was uncovered. Overall, our results provided a sufficient understanding of volunteer tourists’ pro-social decision-making process and behaviors.

## 1. Introduction

An increasing number of travelers have begun to actively seek the assurance that their tourism behaviors have a positive influence on self-enhancement or self-esteem as well as on destination communities and the greater society [1,2]. This has resulted in the rapid growth of volunteer tourism across the globe for the last few decades [3]. Volunteer tourism refers to traveling in an organized way for volunteer activities for various altruistic reasons [4,5]. Volunteer tourism is undeniably the critical facet of altruistic (pro-social) tourism [1], not only offering authentic traveling experiences and emotional/functional values to volunteers, but also contributing to the local destination community directly [3,6,7,8]. Diverse volunteer tourism activities (e.g., education support, environmental protection, medical aid, childcare, ecological restoration, natural disaster recovery assistance) are the critical forms of socially responsible tourism behaviors [3,4,5,9]. This pro-social tourism is often seen as an alternative to other forms of tourism in bringing about psychological benefits to the volunteers [5]. Considering the positive changes in volunteers, the systematic evaluation of how volunteer tourism induces psychological health and pro-social behavioral intention is meaningful for an understanding of volunteering benefits from a volunteer perspective. Researchers agree that the most widely adopted concepts for the comprehension of individuals’ pro-social tourism behaviors (e.g., volunteer tourism activities) are cognitive, affective, and conative factors [10,11,12]. Indeed, existing studies indicated that diverse cognitive and affective factors (e.g., hedonic and utilitarian performances, mental health perception) influence pro-social tourism activities [13,14,15,16]. There has also been a strong support for the crucial role of conative factors (e.g., engagement/involvement) in inducing such altruistic behaviors [17]. Given this, the employment of hedonic and utilitarian performances, mental health, and engagement can be indisputably of utmost criticality for a better comprehension of individuals’ altruistic tourism choices and actions.

Despite the importance of these variables, the structural relationships among the variables in generating volunteer travelers’ pro-social intention and behavior were not well-acknowledged. In addition, the anticipation power of the existing theoretical frameworks for volunteer tourism activities is considered insufficient [2,4]. That is, a sturdy theoretical approach offering clear and comprehensive understanding of individuals’ volunteer tourism intention/behavior has rarely been made. Moreover, an integration of problem awareness and ascribed responsibility into the framework pertinent to volunteer travelers’ decision formation/behavior has been hardly attempted. Undoubtedly, the moderating nature of problem awareness and ascribed responsibility are two key concepts in explicating such pro-social decision/behavior [18,19,20]. In other words, the potential moderating effect of these variables on individuals’ decision formation for volunteer tourism has not been empirically investigated.

Taken together, this study was an attempt (1) to develop a theoretical framework explicating the generation of individuals’ pro-social intention for volunteer tourism by including hedonic and utilitarian performances of volunteer tourism, mental health, and engagement as key concepts; (2) to explore the possible role of hedonic and utilitarian volunteer tourism performance in improving mental health; (3) to uncover the moderating effect of two major constructs in the pro-social sector (problem awareness and ascribed responsibility); (4) to unearth the mediating effect of mental health and volunteer tourism engagement within the hypothesized conceptual framework; and (5) identify the comparative importance among research constructs in generating pro-social intention for volunteer tourism. In the subsequent sections, the literature review was presented. Research methods and results are then reported. Finally, theoretical and practical implications are discussed.

## 2. Literature Review

### 2.1. Hedonic and Utilitarian Performance of Volunteer Tourism

Tourism product performance helps travelers make more favorable decisions for the company [13,15,21]. Tourism product performance mainly comprises hedonic and utilitarian aspects [14,21]. Hedonic performance refers to a traveler’s appraisal of the amount of joy, entertainment, fun, pleasure, and excitement that he/she experiences when consuming a particular tourism product [21]. That is, this concept is related to the assessment of the emotional attributes of the tourism product, which is more affectively driven [14,22]. Meanwhile, utilitarian performance indicates a traveler’s appraisal of the amount of functional benefits derived from the tourism product consumption [21]. In other words, this concept is pertinent to the assessment of the instrumental/practical attributes of the tourism product, which is more cognitively driven [14,22].

The hedonic and utilitarian attributes of a product or service affect patrons’ subsequent purchase decision making process and behavior [14,15,22,23]. These researchers asserted that the hedonic and utilitarian experiences elicit satisfaction evaluation, mental health/well-being, product choice, and loyalty behaviors. Specifically, Kim et al. [23] articulated that the hedonic and utilitarian product performances in restaurants are crucial triggers of customers’ mental health and well-being that subsequently boost their post-purchase intentions. According to Brown [22], these hedonic and utilitarian performances can be important patronage motives among hospitality customers. In the airline lounge sector, Kim et al. [15] exhibited that air travelers’ cognitive, emotional, and sensory assessments of airline lounge performances influence their mental health and their overall evaluation of lounge experiences. In the hotel context, Han and Hyun [13] investigated guest behaviors; their empirical finding showed that green product performance significantly affects guests’ mental health and well-being, and subsequently the loyalty intention. Being grounded on the evidences above, the following hypotheses were posited:

**Hypotheses** **1(H1).** *Hedonic performance of volunteer tourism has a positive impact on mental health.*

**Hypotheses** **2(H2).** *Utilitarian performance of volunteer tourism has a positive impact on mental health.*

### 2.2. Mental Health and Its Role

In the modern society, it is unavoidable that individuals are increasingly struggling with mental fatigue/stress [24,25,26,27]. While the term mental health is a complex concept, it is frequently conceptualized as one’s self-rated assessment of his/her current mental health condition [13,24]. The tourism industry plays a vital role in relieving such mental fatigue/stress [15,28]. Mental health enhancement for travelers thus is the key concern for practitioners in the entire tourism industry [29]. Indeed, boosting travelers’ mental health and well-being perception is increasingly becoming the top priority for every sector of the tourism industry. When travelers perceive that their mental health is enhanced during the use of a tourism product/service, they are likely to become involved in the product/service consumption and build positive intentions for it [15,29].

Extant hospitality and tourism literature supported the criticality of mental health in traveler behavior [15,28,29]. Hwang and Lyu [28] investigated the predictors and outcomes of leisure travelers’ mental health. They found that mental health, which is a significant function of cognitive factors, triggers individuals’ attachment to the leisure product and behavioral intention for it. In the luxury restaurant consumption, Hwang and Hyun [29] demonstrated that patrons’ mental health and well-being perception are based on product performance. The mental health and well-being perception generate a pleasurable product experience, a feeling of attachment to the product, and repurchase intention. Kim et al. [15] examined traveler behaviors’ sensory experiences and mental health in the airline lounge sector. Their empirical result showed that travelers’ mental health perception induces positive satisfaction evaluation, increases travelers’ level of engagement/attachment, and enhances their favorable behavioral intentions. According to this evidence, it can be postulated that if travelers believe that volunteer tourism improves their mental health, their engagement and intention levels for volunteer tourism increase. The following hypotheses were therefore developed:

**Hypotheses** **3(H3).** *Mental health has a positive impact on volunteer tourism engagement.*

**Hypotheses** **4(H4).** *Mental health has a positive impact on pro-social intention for volunteer tourism.*

### 2.3. Volunteer Tourism Engagement

Product engagement is often believed to be a critical concept that positively influences individuals’ purchase decision and behavior [30]. It is a well-recognized variable in the marketing literature to explain consumer behavior. Engagement refers to one’s perceived degree of attachment to the object [31]. The object can be a good/service/company/destination [17]. Consistently, in the present study, volunteer tourism engagement indicates travelers’ perceived attachment level to volunteer tourism activities. When an individual’s engagement level is high, he/she feels strong involvement/absorption in a particular behavior (e.g., consumption of a specific product) [32,33]. Product engagement is hence often interchangeably used with the terms “product involvement” and “product attachment” [31].

When customers make a product choice, product engagement plays an essential role in their choice decision [30,31]. In examining cruise travelers’ behaviors, Chua et al. [17] revealed that cruise tourism engagement exerted a significant influence on travelers’ behavioral intention. Han and Hyun [31] examined travelers’ loyalty generation process. Their empirical result showed that product involvement together with travel motivation and satisfaction significantly elicits traveler loyalty behavior. More recently, in the hotel context, Yu et al. [30] found that product engagement is the important trigger of individuals’ behavioral intention. Their finding also demonstrated the significant mediating effect of engagement in forming intention. Accordingly, we developed the following research hypothesis:

**Hypotheses** **5(H5).** *Volunteer tourism engagement has a positive impact on pro-social intention for volunteer tourism.*

### 2.4. Influence of Problem Awareness and Ascribed Responsibility

Problem awareness and ascribed responsibility are key concepts in pro-social behavior [11,12,16,34,35]. Problem awareness indicates travelers’ consciousness level regarding the diverse serious issues/problems, which harms the local destination or destination community [34,36]. Ascribed responsibility is defined as travelers’ feeling of responsibility for the harmful outcomes of not engaging in pro-social tourism behaviors [18,37]. These two constructs are about how strongly travelers believe that the destination community would be damaging if traveler behavioral choices are not pro-social [12,16].

Many studies indicated that problem awareness and ascribed responsibility are crucial in explicating the traveler pro-social intention generation process [12,35,36,38]. Chen and Tung [38] expressed the significant influence of problem awareness on patrons’ pro-social decision formation. Han et al. [36] asserted that when travelers are highly aware of the problem faced by the local destination and feel responsible for it, they often feel obligation to lessen the problem by practicing pro-social tourism behaviors. Han and Hwang [18] examined convention travelers’ choice behaviors. Their finding revealed that the association between pro-environmental intention and its antecedents are strengthened when travelers are strongly concerned for the ecological problem triggered by the convention industry and they feel responsible for it. These findings are consistent with Schwartz and Howard’s [35] and de Groot and Steg’s [39] interpretation of Schwartz’s [40] theory for pro-social behavior, which indicates that problem awareness and ascribed responsibility fortify the strength between pro-social intention and its determinants. The evidence discussed above implied a significant moderating influence of problem awareness and ascribed responsibility on individuals’ socially responsible decision-making. As a result, the following hypotheses were developed:

**Hypotheses** **6a(H6a).** *Problem awareness significantly moderates the linkage between mental health and pro-social intention for volunteer tourism.*

**Hypotheses** **6b(H6b).** *Problem awareness significantly moderates the linkage between volunteer tourism engagement and pro-social intention for volunteer tourism.*

**Hypotheses** **7a(H7a).** *Ascribed responsibility significantly moderates the linkage between mental health and pro-social intention for volunteer tourism.*

**Hypotheses** **7b(H7b).** *Ascribed responsibility significantly moderates the linkage between volunteer tourism engagement and pro-social intention for volunteer tourism.*

### 2.5. Conceptual Model and Hypotheses

**Figure 1:**
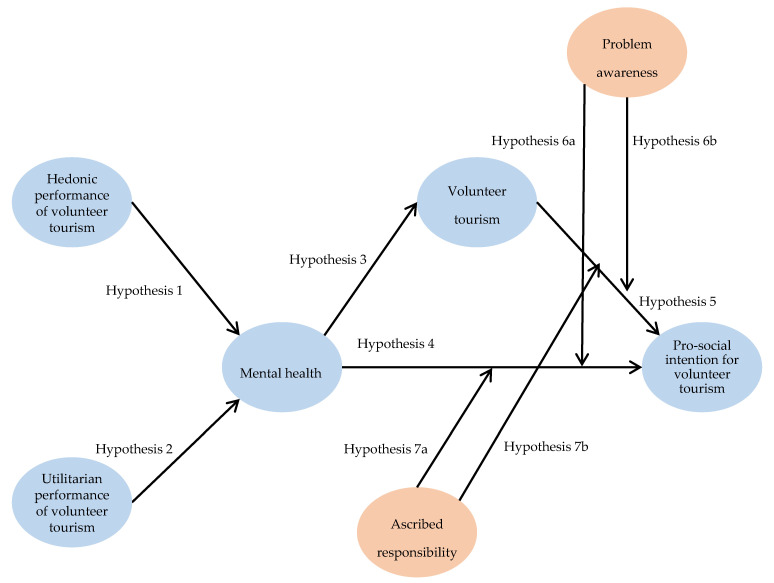
Proposed theoretical model.

Figure 1 exhibits the structural model of this study. The model includes 7 research variables (i.e., hedonic performance, utilitarian performance, mental health, volunteer tourism engagement, problem awareness, ascribed responsibility, pro-social intention for volunteer tourism). In the proposed conceptual framework, problem awareness and ascribed responsibility are the moderators illuminating the structural relationships. Hypotheses 1–5 predicted the causal relationships among study constructs in forming pro-social intention. In addition, Hypotheses 6 and 7 are about the impact of moderators.

## 3. Methodology

### 3.1. Measures and Questionnaire Development

To evaluate research constructs, the measurement items were adopted from the studies in the existing literature [4,10,15,18,21,34,35,41,42]. The multi-items with seven-point scale ranging from “strongly disagree” (1) to “strongly agree” (7) were utilized for the assessment of every construct. Specifically, we used four items to measure hedonic performance of volunteer tourism (e.g., “Being a volunteer has been entertaining and fun”) and four items for utilitarian performance (e.g., “I know that I have helped the host community by being involved in volunteer tourism”) of volunteer tourism. A total of five items were used to assess mental health (e.g., “In most aspects, my life has become close to my ideal after participating in this volunteer tourism program”). In addition, four items for volunteer tourism engagement (e.g., “Anything that is related to this volunteer activity captures my attention”) and three items for problem awareness (e.g., “I have increased my awareness of helping others by participating in this volunteer tourism program”) were utilized. Lastly, three items for ascribed responsibility (e.g., “Helping others through volunteer tourism is my responsibility”) and three items for pro-social intention (e.g., “I will exert an effort to participate again in a volunteer tourism program in the near future”) were used. The survey questionnaire included these measurement items along with the research description. The initial version of the questionnaire was pre-tested using content validity method. In particular, ten tourism academics participated in this pre-test. A minor improvement on the questionnaire was made through this procedure. Subsequently, a pilot-test was performed with 89 students whose major is tourism management. The survey questionnaire was then perfected by two professors in the tourism management department. The original version of the questionnaire was in English. It was translated into Korean by employing a back-to-back method. The details of the measures are presented in Table A1 (Appendix A). 

Because of the observational nature of the study, and in the absence of any involvement of therapeutic medication, no formal approval of the Institutional Review Board of the local Ethics Committee was required. Nonetheless, all subjects were informed about the study and participation was fully on voluntary basis. The study was conducted in accordance with the Helsinki Declaration.

### 3.2. Data Collection Process

The data were collected during the Goodnews Corp Festival in Spring, 2019. The festival was non-profit where many volunteer tourists attended. Specifically, travelers who voluntarily completed the non-profit global volunteer tourism program along with their family members were the major participants of the festival. Our survey questionnaire was distributed to the volunteer tourists during the break time of the festival. All tourists participated in the survey in a voluntary manner. They were asked to recall their volunteer tourism experiences and to fill out the questionnaire. The completed questionnaires were returned onsite. The surveyor also checked the completeness of the questionnaire onsite. As a token of appreciation, a USB, which was worth about US$30.00, was given to the respondents. After excluding unusable cases, a total of 350 usable responses were obtained through this procedure.

### 3.3. Data Analysis

The study utilized Anderson and Gerbing’s [43] two-step approach to evaluate the model and to examine the research hypotheses. More specifically, the adequacy of the measurement model was tested using confirmatory factor analysis. Next, the hypothesized paths within the structural model were evaluated through structural equation modeling. Moreover, the moderating effects of problem awareness and ascribed responsibility were assessed through a metric invariance test.

## 4. Results

### 4.1. Socio-Demographic Characteristics of Sample

Of 350 respondents, 54% were female volunteer travelers and 46.0% were male volunteer travelers. Their age ranged from 20 to 37 years old. Their mean age was 23.87 years old. Regarding their household income, about 51.9% reported the income between $40,000–$69,999, followed by $25,000 or less (30.7%), and $70,000 or more (17.4%). In terms of the participants’ education level, about 78.9% reported that they have completed (or currently enrolled in) four-year college (78.9%), followed by two-year college or less (18.0%) and graduate degree holders (3.1%). Regarding the length of the participants’ volunteer tourism, about 60.9% indicated five to six months, followed by one year or longer (34.9%), three to four months (4.3%), and one to two months (2.0%). The frequency of volunteer tourism was asked. The majority of the participants reported once (83.1%), followed by twice (7.4%), four times or more (6.0%), and three times (3.4%).

### 4.2. Measurement Quality Assessment

Before evaluating the proposed model, a confirmatory factor analysis was conducted for the assessment of the measurement quality. Results indicated that the model including all measurement items contained a satisfactory goodness-of-fit statistics (χ^2^ = 759.229, *df* = 277, *p* < 0.001, χ^2^/*df* = 2.741, RMSEA = 0.071, CFI = 0.925, IFI = 0.925, TLI = 0.912). Composite reliability was calculated. Our calculation revealed that all values (hedonic performance = 0.900; utilitarian performance = 0.852; mental health = 0.861; volunteer tourism engagement = 0.853; problem awareness = 0.825; ascribed responsibility = 0.834; pro-social intention = 0.953) are above the recommended threshold of 0.700 [44] (see Table 1). Thus, internal consistency of the multiple-item measures was demonstrated. Average variance extracted values for study constructs were subsequently calculated. Our results showed that the values (hedonic performance = 0.694; utilitarian performance = 0.599; mental health = 0.557; volunteer tourism engagement = 0.594; problem awareness = 0.612; ascribed responsibility = 0.628; pro-social intention = 0.870) are all above the suggested cutoff of 0.500 [44]. In addition, the average variance extracted values were greater than the between-factor correlations (squared) (see Table 1). Hence, convergent validity for research variables was evident.

### 4.3. Structural Model Assessment

**Figure 2:**
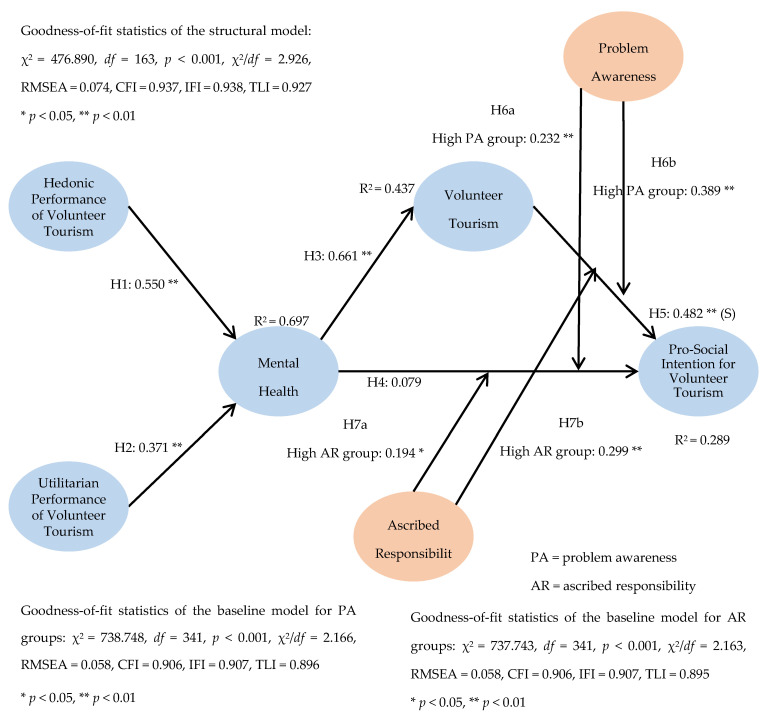
Structural model and baseline model estimations (n = 350).

A structural equation modeling was conducted. Result showed that the model contained a satisfactory level of goodness-of-fit statistics (χ^2^ = 476.890, *df* = 163, *p* < 0.001, χ^2^/*df* = 2.926, RMSEA = 0.074, CFI = 0.937, IFI = 0.938, TLI = 0.927). The proposed theoretical framework accounted for about 69.7%, 43.7%, and 28.9% of the total variance in mental health, volunteer tourism engagement, and pro-social intention, respectively. The hypothesized effect of volunteer tourism performance was tested. As shown in Table 2 and Figure 2, the hedonic performance of volunteer tourism (β = 0.550, *p* < 0.01) and utilitarian performance of volunteer tourism (β = 0.371, *p* < 0.01) exerted a significant influence on mental health. This result supported Hypotheses 1 and 2. The effect of mental health was assessed. Result showed that mental health had a positive and significant influence on volunteer tourism engagement (β = 0.661, *p* < 0.01). Yet, the relationship between mental health and pro-social intention was not significant. Therefore, while Hypothesis 3 was supported, Hypothesis 4 was not supported. The association between volunteer tourism engagement and pro-social intention was tested. Our result indicated that volunteer tourism engagement exerted a significant effect on pro-social intention (β = 0.482, *p* < 0.01). Hence, Hypothesis 5 was supported. Table 2 and Figure 2 contained the details regarding the structural model assessment results. Indirect effect of research constructs was evaluated. As reported in Table 2, both hedonic performance (β = 0.363, *p* < 0.01) and utilitarian performance (β = 0.245, *p* < 0.01) had a significant indirect influence on volunteer tourism engagement. In addition, hedonic performance (β = 0.219, *p* < 0.01), utilitarian performance (β = 0.147, *p* < 0.05), and mental health (β = 0.319, *p* < 0.01) had a significant indirect impact on pro-social intention for volunteer tourism. This result implies that mental health and volunteer tourism engagement played an important mediating role in the proposed theoretical framework. Subsequently, the total effect of study variables was assessed. As reported in Table 2, volunteer tourism engagement had the greatest total effect on pro-social intention (β = 0.482, *p* < 0.01), followed by mental health (β = 0.398, *p* < 0.01), hedonic performance (β = 0.219, *p* < 0.01), and utilitarian performance (β = 0.147, *p* < 0.01).

### 4.4. Baseline Model Assessment and Invariance Test

A test for metric invariance was conducted in order to evaluate the moderating influence of problem awareness and ascribed responsibility. The participants’ responses for problem awareness and ascribed responsibility were split into high and low groups with the use of K-means cluster analysis. The high group of problem awareness contained 269 responses, whereas the low group of problem awareness included 81 responses. In addition, the high group of ascribed responsibility contained 231 responses, whereas the low group of ascribed responsibility included 119 responses. Baseline models in which all factor loadings were restricted to be equivalent between high and low groups were generated. Our result showed that the baseline model for problem awareness groups (χ^2^ = 738.748, *df* = 341, *p* < 0.001, χ^2^/*df* = 2.166, RMSEA = 0.058, CFI = 0.906, IFI = 0.907, TLI = 0.896) and the baseline model for ascribed responsibility groups (χ^2^ = 737.743, *df* = 341, *p* < 0.001, χ^2^/*df* = 2.163, RMSEA = 0.058, CFI = 0.906, IFI = 0.907, TLI = 0.895) had an adequate level of goodness-of-fit statistics. The details about the baseline model assessment results are shown in Table 3 (model for problem awareness groups), Table 4 (model for ascribed responsibility groups), and Figure 2.

Subsequently, a chi-square test was performed. The baseline models were compared to nested models where a particular path of interest is restricted to be equal. Our result showed that the mental health and pro-social intention relationship was significantly different across high and low groups of problem awareness (Δχ^2^ [1] = 6.152, *p* < 0.05). Therefore, Hypothesis 6a was supported. However, the relationship between volunteer tourism engagement and pro-social intention was not significantly different between high and low groups of problem awareness (Δχ^2^ [1] = 0.045, *p* > 0.05). Thus, Hypothesis 6b was not supported. Next, the mental health and pro-social intention relationship for ascribed responsibility groups was tested. Our result showed the significant difference across two groups (Δχ^2^ [1] = 6.237, *p* < 0.05). This result supported Hypothesis 7a. Yet, the relationship between volunteer tourism engagement and pro-social intention was not significantly different between high and low groups of ascribed responsibility (Δχ^2^ [1] = 3.151, *p* > 0.05). Thus, Hypothesis 7b was not supported.

## 5. Discussion

As proposed in the present research, it was expected that hedonic and utilitarian performances of volunteer tourism would determine travelers’ mental health. This finding supports past studies regarding product/service performance as an important variable explaining mental health [13,15,23]. Meeting the expectation, our result demonstrated that hedonic and utilitarian performances of volunteer tourism significantly and positively contributed to increasing travelers’ mental health, which ultimately brings the enhanced pro-social intention. Given the result of this research, it is essential to boost volunteer tourism performance. In particular, volunteer tourism destination/program practitioners should deal with the hedonic facet of volunteer tourism. For instance, developing and distributing positive songs/messages that boost feelings of pride, fulfillment, and pleasure while volunteering that increase a sense of accomplishment can be of importance in improving travelers’ affective experiences. Practitioners should also provide visitors with functional/instrumental benefits (e.g., personal development opportunity, escapism, cultural experiences, self-enhancement). Based on these efforts, volunteer travelers’ mental health perception can be improved.

Our finding demonstrated that problem awareness moderated the influence of mental health on pro-social intention for volunteer tourism, which is consistent with past studies identifying the moderating role of problem awareness in pro-social decision formation [36,38]. Specifically, the strength of the relationship between mental health and pro-social intention for volunteer tourism was more intense in the high problem awareness group than in the low group (high: β = 0.232, *p* < 0.01 vs. low: β = −0.129, *p* > 0.05). The result from the metric invariance test suggests that at the similar level of mental health, volunteer tourists with high problem awareness build a stronger pro-social intention than those with low problem awareness. That is, boosting mental health more likely leads to the increased pro-social intention among volunteer tourists who are strongly aware that people at volunteer tourism destinations need assistance.

Regarding the role of ascribed responsibility, our findings provided evidence that this variable moderated the effect of mental health on pro-social intention for volunteer tourism. This finding reinforces the important role of ascribed responsibility in pro-social activities [18,39]. In particular, the strength of the relationship between mental health and pro-social intention for volunteer tourism was greater in the high ascribed responsibility group than in the low group (high: β = 0.194, *p* < 0.05 vs. low: β = −0.171, *p* > 0.05). This finding from the invariance test implies that at the similar level of ascribed responsibility, volunteer tourists with high ascribed responsibility have a stronger pro-social intention than those with low ascribed responsibility. In other words, increasing ascribed responsibility more likely results in the enhanced pro-social intention among tourists who have a strong belief that helping local communities at volunteer tourism sites is everyone’s responsibility.

Our empirical evidence about the moderating effect of such variables has important theoretical and practical meanings. Theoretically, the present research is the first to demonstrate the moderating nature of problem awareness and ascribed responsibility in explicating volunteer tourists’ pro-social decision-making process. Integrating these variables into diverse theory and conceptual model developments as moderators can be effective for the clear explanation of various volunteer tourism activities. From the practical manner, volunteer tourism practitioners should never neglect such differences between high and low groups of problem awareness and ascribed responsibility when developing new strategies (or fortifying the existing ones) for maximizing volunteer tourists’ pro-social behaviors (e.g., repeated participation in a volunteer tourism program, helping the host community). An increase of volunteers’ awareness regarding local people’s and community’s needs for help and an increase of volunteers’ feelings of responsibility for filling such needs are essential for the efficient elicitation of volunteer tourists’ pro-social intention/behavior. Practitioners therefore should actively help volunteer tourists know about the serious issues of volunteer tourism destinations and local people (e.g., natural disasters, diseases, childcare, education, community development, protection of animals) through various communication channels (e.g., TV, Internet, magazines, SNS). This effort would ultimately contribute to strengthening existing/potential volunteer tourists’ problem awareness and ascribed responsibility.

The prominent role of volunteer tourism engagement in boosting pro-social intention was uncovered. According to Fisher’s z-test result, its total influence was significantly higher than the other research constructs (*p* < 0.01). This result contains a critical theoretical meaning as it provides evidence that the nature of pro-social intention formation among volunteer tourists includes involvement/flow process through rigorous empirical procedure. According to Han et al. [4], dealing with individuals’ belief that their volunteering really makes a difference is of utmost criticality in increasing their engagement level for volunteer tourism. Therefore, practically, helping volunteer tourists to know their volunteering and its direct/potential contributions to the destination community and people (e.g., making a difference to the lives of local people/animals and to the natural environment) can be an excellent way for the effectual stimulation of individuals’ volunteer tourism engagement that directly leads to the pro-social intention enhancement.

In this research, mental health and volunteer tourism engagement played a significant mediating role in forming volunteer travelers’ pro-social intention. This finding sustained the view that mental health and product engagement maximize the influence of their predictor(s) on their outcome factor(s). Volunteer tourism researchers should understand that volunteer tourism performance—pro-social intention relationship is intervened when mental health and engagement are involved in a theoretical framework. Understanding the mediating characteristics of these concepts is critical for scholars to utilize mental health and product engagement when developing a sturdy theorization associated with traveler pro-social decision-making process for volunteer tourism. For practitioners, in order to magnify the influence of volunteer tourism performance on travelers’ positive decision, focusing on the improvement of mental health and product engagement can be essential.

## 6. Limitations and Recommendations for Future Research

Despite the essential meaning in theory/practice, the present research includes several limitations. First, many barriers (e.g., time, risk, money, skills) can exist when individuals make volunteer tourism choices. Undoubtedly, the barriers would significantly play a role in volunteer tourists’ decision-making process. Nonetheless, the present study did not take the possible effect of the barriers into consideration. Future studies should pinpoint the possible barriers of volunteer tourism choice and apply them into our proposed conceptual framework. This broadening process can improve the comprehensiveness of the proposed model and strengthen its prediction power. Second, since this study centered on individuals’ volunteer tourism choice decision in general, specific volunteer tourism activities taking place in the local destination were not further examined. Future research should explore the types of volunteer tourism activities and investigate if the uncovered types of behaviors are under the influence of the driving factors identified in the present study. Third, the survey was conducted at the Goodnews Corp Festival in South Korea. Future studies are recommended to examine volunteer tourism experiences from volunteers in different volunteer organizations or programs. This will help increase the validity of the conceptual model.

## 7. Conclusions

This present research investigated the pro-social intention generation process among international volunteer tourists by employing hedonic and utilitarian performances of volunteer tourism, mental health, volunteer tourism engagement, problem awareness, and ascribed responsibility as the key concepts. The hypothesized paths within the proposed conceptual model were statistically supported. Volunteer tourism engagement was the most critical factor in determining pro-social intention. In addition, it was uncovered that problem awareness and ascribed responsibility fortified the effect of volunteer tourism engagement on intention. In the global volunteer tourism marketplace, inducing individuals’ pro-social decision for repeat participation in a volunteer tourism program and for helping the volunteer tourism destination community is essential in order to make the destination a better place for the lives of local residents/habitats. Our findings can be useful when researchers and practitioners develop effective strategies for triggering such pro-social decision. Moreover, this research contributed to the scarce literature on volunteer tourists’ post-experience behaviors by demonstrating the active role of hedonic and utilitarian aspects of volunteer tourism performance, mental health, engagement, and the moderating role of problem awareness and ascribed responsibility.

## Figures and Tables

**Table 1:** Measurement model assessment.

Variables (loadings) (R^2^)	1	2	3	4	5	6	7	AVE(CR)
1. Hedonic performance of volunteer tourism (0.877, 0.910, 0.733, 0.800) (0.769, 0.828, 0.537, 0.640)	1.000	–	–	–	–	–	–	0.694 (0.900)
2. Utilitarian performance of volunteer tourism (0.831, 0.919, 0.764, 0.527) (0.691, 0.845, 0.583, 0.277)	0.580 ^a^ (0.336) ^b^	1.000	–	–	–	–	–	0.599 (0.852)
3. Mental health (0.635, 0.765, 0.685, 0.816, 0.812) (0.404, 0.585, 0.469, 0.666, 0.660)	0.686 (0.471)	0.634 (0.402)	1.000	–	–	–	–	0.557 (0.861)
4. Volunteer tourism engagement (0.721, 0.709, 0.848, 0.797) (0.520, 0.503, 0.719, 0.635)	0.465 (0.216)	0.503 (0.253)	0.567 (0.321)	1.000	–	–	–	0.594 (0.853)
5. Problem awareness (0.682, 0.807, 0.849) (0.465, 0.651, 0.721)	0.518 (0.268)	0.587 (0.345)	0.642 (0.412)	0.408 (0.166)	1.000	–	–	0.612 (0.825)
6. Ascribed responsibility (0.757, 0.735, 0.878) (0.573, 540, 0.777)	0.451 (0.203)	0.449 (0.202)	0.519 (0.269)	0.470 (0.221)	0.569 (0.324)	1.000	–	0.628 (0.834)
7. Pro-social intention for volunteer tourism (0.921, 0.963, 0.914) (0.849, 0.927, 836)	0.249 (0.062)	0.322 (0.104)	0.365 (0.133)	0.502 (0.252)	0.262 (0.069)	0.390 (0.152)	1.000	0.870 (0.953)
Mean	6.306	6.284	6.253	5.624	6.312	5.982	5.191	–
Standard deviation	0.869	0.791	0.802	1.198	0.784	0.983	1.752	–

Note. Goodness-of-fit statistics: χ^2^ = 759.229, *df* = 277, *p* < 0.001, χ^2^/*df* = 2.741, RMSEA = 0.071, CFI = 0.925, IFI = 0.925, TLI = 0.912.; ^a^ Correlations between variables are below the diagonal.; ^b^ Squared correlations between variables are within parentheses.

**Table 2:** Results of structural equation modeling (n = 350).

Hypothesized Paths	Coefficients	t-Values
H1: Hedonic performance → Mental health	0.550	9.127 **
H2: Utilitarian performance → Mental health	0.371	5.773 **
H3: Mental health → Volunteer tourism engagement	0.661	9.634 **
H4: Mental health → Pro-social intention for volunteer tourism	0.079	1.047
H5: Volunteer tourism engagement → Pro-social intention for volunteer tourism	0.482	6.026 **
Indirect effect on pro-social intention for volunteer tourism: β_Mental health_ = 0.319 ** β_Hedonic performance_ = 0.219 ** β_Utilitarian performance_ = 0.147 *	Total effect on Pro-social intention for volunteer tourism: β_Volunteer tourism engagement_ = 0.482 ** β_Mental health_ = 0.398 ** β_Hedonic performance_ = 0.219 ** β_Utilitarian performance_ = 0.147 *	Goodness-of-fit statistics: χ^2^ = 476.890, *df* = 163, *p* < 0.001, χ^2^/*df* = 2.926, RMSEA = 0.074, CFI = 0.937, IFI = 0.938, TLI = 0.927
Indirect effect on volunteer tourism engagement: β_Hedonic performance_ = 0.363 ** β_Utilitarian performance_ = 0.245 **	Total variance explained: R^2^ (pro-social intention for volunteer tourism) = 0.289 R^2^ (volunteer tourism engagement) = 0.437 R^2^ (mental health) = 0.697	

* *p* < 0.05, ** *p* < 0.01.

**Table 3:** Results of structural invariance model - problem awareness.

Paths	High PA Group (n = 269)		Low PA Group (n = 81)		Baseline Model (Freely Estimated)	Nested Model (Constrained to Be Equal)
	β	t-Values	β	t-Values		
H6a: Mental health → Pro-social intention for volunteer tourism	0.232	2.777 **	−0.129	−0.816	χ^2^ (341) = 738.748	χ^2^ (342) = 744.900 ^a^
H6b: VT engagement → Pro- social intention for volunteer tourism	0.389	4.584 **	0.542	3.349 **	χ^2^ (341) = 738.748	χ^2^ (342) = 738.793 ^b^
Chi-square difference test: ^a^ Δχ^2^ (1) = 6.152, *p* < 0.05 ^b^ Δχ^2^ (1) = 0.045, *p* > 0.05	Hypotheses testing: H6a: Supported H6b: Not supported			Goodness-of-fit statistics: χ^2^ = 738.748, *df* = 341, *p* < 0.001, χ^2^/*df* = 2.166, RMSEA = 0.058, CFI = 0.906, IFI = 0.907, TLI = 0.896		

Note. PA = problem awareness, VT = volunteer tourism; * *p* < 0.05, ** *p* < 0.01.

**Table 4:** Results of structural invariance model—ascribed responsibility.

Paths	High AR Group (n = 231)		Low AR Group (n = 119)		Baseline Model (Freely Estimated)	Nested Model (Constrained to Be Equal)
	β	t-Values	β	t-Values		
H7a: Mental health →Pro-social intention for volunteer tourism	0.194	2.245 *	−0.171	−1.253	χ^2^ (341) = 737.743	χ^2^ (342) = 743.980 ^a^
H7b: VT engagement → Pro-social intention for volunteer tourism	0.299	3.455 **	0.585	3.965 **	χ^2^ (341) = 737.743	χ^2^ (342) = 740.894 ^b^
Chi-square difference test: ^a^ Δχ^2^ (1) = 6.237, *p* < 0.05 ^b^ Δχ^2^ (1) = 3.151, *p* > 0.05	Hypotheses testing: H7a: Supported H7b: Not supported			Goodness-of-fit statistics: χ^2^ = 737.743, *df* = 341, *p* < 0.001, χ^2^/*df* = 2.163, RMSEA = 0.058, CFI = 0.906, IFI = 0.907, TLI = 0.895		

Note. AR = ascribed responsibility, VT = volunteer tourism; * *p* < 0.05, ** *p* < 0.01

## Appendix A

**Table A1:** Constructs and measurement items.

**Hedonic Performance of Volunteer Tourism**
● Being a volunteer has been entertaining and fun. ● Being a volunteer has been an exciting and moving experience. ● A good atmosphere has been established among the volunteers. ● I have been excited to work for a VT project with a universal character.
**Utilitarian Performance of Volunteer Tourism**
● I know that I have helped the host community by being involved in VT. ● VT exerts a positive impact on the host community. ● VT is a positive accomplishment for me. ● VT is good addition to my resume and job applications.
**Mental Health**
● In most aspects, my life has become close to my ideal after participating in this VT program. ● Thus far, I have obtained the important things I want in my life after participating in this VT program. ● If I can live my life over, then I will change nearly nothing after participating in this VT program. ● In general, I consider myself very happy after participating in this VT program. ● I am generally very happy and I enjoy life after participating in this VT program.
**Volunteer Tourism Engagement**
● Anything that is related to this volunteer activity captures my attention. ● When I am interacting during this activity, I forget everything else around me. ● I am immersed in my interactions during this activity. ● In general, I like to get involved in discussions during this activity.
**Problem Awareness**
● Through this VT program, I have become aware that people need help. ● I have increased my awareness of helping others by participating in this VT program. ● My behavior in this VT program can help people and communities.
**Ascribed Responsibility**
● Helping others through VT is my responsibility. ● I can contribute to helping other people/local communities. ● I should be responsible for helping others through a VT program.
**Pro-social Intention for Volunteer Tourism**
● I am planning to participate again in a VT program in the near future. ● I will exert an effort to participate again in a VT program in the near future. ● I am willing to participate again in a VT program in the near future.

Note. All measurement items except for the items for attitude toward eating were evaluated with a seven-point scale from “strongly disagree” (1) to “strongly agree” (7).
